# The Value of Companion Dogs as a Source of Social Support for Their Owners: Findings From a Pre-pandemic Representative Sample and a Convenience Sample Obtained During the COVID-19 Lockdown in Spain

**DOI:** 10.3389/fpsyt.2021.622060

**Published:** 2021-04-14

**Authors:** Jonathan Bowen, Antonio Bulbena, Jaume Fatjó

**Affiliations:** ^1^Affinity Foundation Chair for Animals and Health, Department of Psychiatry and Forensic Medicine, School of Medicine, Autonomous University of Barcelona, Barcelona, Spain; ^2^Queen Mother Hospital for Small Animals, Royal Veterinary College, North Mymms, United Kingdom; ^3^Institut Hospital del Mar d'Investigacions Mèdiques - Institut de Neuropsiquiatria i Addiccions, Mar Health Park, Barcelona, Spain

**Keywords:** social support, crisis, dog, bereavement, COVID-19

## Abstract

Dogs are a source of companionship and comfort for their owners, but the degree to which this might translate into real emotional and social support has not been quantified. Emotional and social support are essential to help people to get through personal crises such as bereavement. In this study we characterize the social support owners obtain from their dogs, provide evidence of how widespread this social support is amongst dog-owners, and show how social support from dogs can increase during a crisis (using the COVID-19 pandemic as an example). We collected data from a representative population-based sample of Spanish dog-owners and found that most respondents said that their dogs helped them to get through tough times. They got comfort from physical contact with their dogs, shared activities with them and treated them as confidants in a similar way to friends and family. These are all key aspects of social support, and dogs offer the advantage of being more available than human sources of support. It would be expected that the support that dogs provide would be increased during a time of personal crisis and when we looked at data collected from a convenience sample of Spanish dog-owners during the COVID-19 confinement that is what we found; during the confinement owners engaged in more shared activities with their dogs, hugged them more often and turned to them more as a source of companionship and comfort (*p* < 0.0001 in all cases). However, although owners did confide more in their dogs (*p* < 0.0001), the effect was not as great as for other aspects of social support. We suspect that this is because people were able to use telecommunications such as video conferencing to maintain their human confidant relationships. Our findings indicate that dogs can substitute for humans as sources of some kinds of social support when conventional sources are unavailable. Our conclusion is that where a dog is present in a household, it should be regarded as an important resource for social support. This should be considered when designing clinical interventions and when public health decisions are being made.

## Introduction

A recent systematic review suggests that the COVID-19 pandemic is having significant psychological effects on the general populations of many countries including the USA, China, Italy and Spain (1). In particular, the pandemic has had a substantial effect on both the prevalence of grief and its management. The process of grieving has been negatively affected by reduced social interactions due to restrictions and lockdowns, financial insecurity, fear of contagion, and the limitations on the holding of funerals and burials that have resulted from pandemic control measures (2). The pandemic is also having detrimental effects on positive lifestyle factors, such as physical activity and healthy dietary habits (3).

There is a very robust relationship between access to social support and positive indicators of physical and mental health (4), and social support has been one of the main protective factors against stress and anxiety during the pandemic (1). The stress-buffering hypothesis proposes that social support acts as a buffer to reduce the negative impact of stressful situations on health and well-being, as well as to compensate individual trait vulnerabilities, such as neuroticism and introversion (5, 6). However, during the pandemic there has been a profound disruption of social support networks and access to regular face-to-face medical services, including counseling and psychological support to the bereaved, which has exacerbated existing vulnerabilities (7). This is a situation in which pet dogs could become a supplementary source of support, not only in the face of highly stressful events, but also as a buffer to help people to cope with the background of every day low-level stressors.

Social support is an overarching construct that includes several somewhat independent components. Two of the main features of any given source of social support are availability and closeness (4). Availability relates to how easily a source of social support can be accessed, and closeness includes interdependence, shared activities and emotional support (8). Another valuable role in a relationship is that of “confidant;” someone with whom to share personal thoughts. Self-disclosure is the main characteristic of a confidant relationship (9). Physical contact is also important to social support, and has positive effects on socio-emotional, physical and psychological well-being (10). Although not part of social support, for many people there is a psychological benefit to having opportunities to care for others (11).

According to recent estimates, 24% of European households own at least one dog (12) and most people who live with dogs consider them to be like family members (13). Also, several studies have found neurophysiological similarities between human-dog relationships and interactions between people, particularly between the mothers and their children (14). Previous research has identified pets as one of the potential sources of social support that could contribute to mental resilience (6, 15, 16). However, to the best of our knowledge no previous research has explored how companion dogs might fit into the multi-faceted framework of social support, in the general population.

In this study we sought to confirm our hypotheses that the social support people get from their companion dogs is a widespread phenomenon, that the types of support that people get from their dogs is comparable in character to that which is obtained from people, and that social support from dogs would increase during a time of crisis.

## Methods

Two samples of data were collected in Spain. The main focus of the study was a representative sample population that was obtained in early 2019, a year before the COVID-19 pandemic. This data was used to establish the types and levels of support that dog owners would be expected to obtain from their pets. A second, convenience sample, was collected during the initial period of the COVID-19 pandemic lockdown in Spain. This data was used to provide evidence for the effect that a personal crisis might have on the degree to which people seek such social support from pets. The data from the representative sample population was collected anonymously, so it was not possible to re-contact the respondents during the pandemic. For both populations, a questionnaire based on the Monash Dog Owner Relationship Scale (17) (MDORS) was used to evaluate the social support obtained by the owner from the pet dog. Data was analyzed at the level of the individual item scores for the scales that were used, as the subscale scores for these scales were originally designed to measure the human-animal bond, and include items that are not relevant to social support.

### Spanish Representative Sample Population

Five hundred and one responses from dog owners were collected in Spain during March-April 2019 by Ipsos MORI for the Affinity Foundation Chair for Animals and Health and the Affinity Foundation. Data were collected from an Ipsos Panel representative sample of the population of Spanish dog owners. The sample population included dog owners who were adult Spanish residents (above 18 years of age). The sampling method applied national representative quotas related to the socio-demographic features of the owner and general characteristics of the dog. Data was obtained following a standard CAWI (Computer Assisted Web Interviewing) procedure. Respondents completed demographic questions about themselves and their history of dog ownership, as well as the MDORS (17). A previously prepared, standardized, back-translated Spanish language version of MDORS questionnaire was used (18). In the conventional scoring of MDORS, the items within the perceived costs subscale are reverse scored. This means that a high score for the perceived costs subscale equates to a low-level of perceived costs. Although this is confusing, it means that high scores for all three subscales indicate a better human-animal bond, and a single value for quality of relationship can be calculated by combining the scores from the subscales. This was not relevant to our study, so to reduce confusion all items were scored in the same way (none were reverse scored). For example, “Strongly agree” was scored 5 for all items, regardless of which subscale they originated from. This makes it easier to interpret the results, within the context of the present study, particularly in combination with the data from the COVID lockdown convenience sample.

### Spanish COVID Convenience Sample Population

Two weeks after the start of the COVID lockdown in Spain, an online questionnaire about the impact of the lockdown on pets and household was distributed through social media of the Affinity Foundation Chair for Animals and Health, the Affinity Foundation, AVEPA/VetBonds (Asociación de Veterinarios Españoles Especialistas en Pequeños Animales), the Grupo de Especialidad de Etología Clínica de Avepa (GrETCA), the Fundación para el Asesoramiento y Acción en Defensa de los Animales (FAADA), Single Track Ltd, and veterinary clinics associated with AVEPA. The lockdown included the following measures; social distancing, the closure of schools and universities, banning of mass gatherings and public events, and the suspension of all non-essential economic activities (19). Data collection ended after 2 weeks. The survey was hosted on the SurveyGizmo® platform (which is now known as Alchemer®). Seven hundred and ninety-four responses were collected from dog owners (no data from cat owners was included).

Respondents completed a modified version of the Cat/Dog-Owner Relationship scale (C/DORS), that was developed from the MDORS by the authors, for the measurement of the human-animal bond between owners and their cats or dogs (20). The differences between MDORS and C/DORS mostly relate to items from the owner-pet interaction sub-scale, because, for example, cat owners don't usually take their cats with them to visit friends and family. Three items were excluded from this part of the study, either because they were in direct contravention of the lockdown regulations (“How often do you take your dog to visit people?” and “How often do you take your dog in the car?”), or because they had the potential to cause substantial distress to people during a time of crisis (“How traumatic do you think it will be for you when your dog dies?”). Scoring of the items was also different from the standard implementation of C/DORS or MDORS: All items were scored on a 5-point scale from “Much more than before the confinement,” which was scored +2, to “Much less than before the confinement,” which was scored −2. One reason for changing the scoring system was that, as we will see, scores for many items of MDORS were already maximal for most respondents in the representative sample. A scale from “much more” to “much less” than before the COVID confinement not only restored the range for each item, but also made it clear to respondents that they were being asked to compare their current situation (during the lockdown confinement) with prior to the lockdown.

### Statistics

Data was tabulated, mean scores and other descriptive statistics were calculated in Microsoft Excel (version 16. 2020). After finding that data was not normally distributed (D'Agostino-Pearson test), Spearman rank correlation was used to investigate correlations between variables in the representative sample. A Bonferroni correction was applied to these correlations, so that the threshold for significance was 0.0026 (0.05 divided by 19).

For the convenience sample COVID-19 population, a single-sample Wilcoxon Signed Rank Test was used to determine whether responses for each modified MDORS item were significantly different from the mid-point response “The same as before the confinement.” A Bonferroni correction was applied to those contrasts, so that the threshold for significance was 0.002 (0.05 divided by 25). Statistical tests were performed using Prism version 8 (Graphpad Software, 2020).

## Results

### Spanish Representative Sample Population

The mean age of the 501 respondents was 41.7 years of age, with 47.9% being male and 52.1% being female. The mean number of dogs per household was 1.3 (SD 0.6). A table of the individual item scores is presented in Table 1, with the items sorted according to their location in the three MDORS sub-scales (interaction, emotional closeness and perceived costs).

**Table 1:** Individual item scores for MDORS items (representative sample).

				**Scores (%)**						
		**Mean score**	**SD**	**5**	**4**	**3**	**2**	**1**	**% scoring 4 or 5**	**Meaning of score 4 or 5**
Interaction	How often do you play games with your dog?	4.7	0.7	79.8	14.8	3.0	1.4	1.0	94.6	At least once a day or once every few days
	How often do you have your dog with you while relaxing, ie watching TV?	4.5	1.0	77.4	11.0	4.8	1.8	5.0	88.4	At least once a day or once every few days
	How often do you hug your dog?	4.5	1.0	71.0	15.8	5.8	3.6	3.8	86.8	At least once a day or once every few days
	How often do you kiss your dog?	3.7	1.7	56.9	12.2	3.4	3.0	24.5	69.1	At least once a day or once every few days
	How often do you give your dog food treats?	3.5	1.3	27.7	29.1	21.0	11.0	11.2	56.8	At least once a day or once every few days
	How often do you take your dog to visit people?	3.5	1.5	37.9	18.8	18.1	6.0	19.2	56.7	Once a week or once a fortnight
	How often do you groom your dog?	3.4	1.1	15.6	39.1	21.5	16.6	7.2	54.7	At least once a day or once every few days
	How often do you take your dog in the car?	2.9	1.2	9.0	26.9	24.6	27.5	12.0	35.9	At least once a day or once every few days
	How often do you buy your dog presents?	3.0	1.0	7.2	22.0	37.5	27.3	6.0	29.2	Once a week or once a fortnight
Emotional closeness	If everyone else left me my dog would still be there for me.	4.6	0.7	69.8	20.8	8.6	0.8	0.0	90.6	Strongly agree or agree
	My dog provides me with constant companionship.	4.6	0.7	67.2	23.0	8.4	1.0	0.4	90.2	Strongly agree or agree
	How traumatic do you think it will be for you when your dog dies?	4.4	0.8	58.7	28.7	10.4	1.2	1.0	87.4	Very traumatic or traumatic
	My dog is there whenever I need to be comforted.	4.5	0.8	60.9	26.1	11.0	1.4	0.6	87.0	Strongly agree or agree
	I wish my dog and I never had to be apart.	4.4	0.8	58.5	26.7	11.2	3.0	0.6	85.2	Strongly agree or agree
	My dog helps me get through tough times.	4.3	0.9	50.7	29.3	16.2	2.8	1.0	80.0	Strongly agree or agree
	My dog gives me a reason to get up in the morning.	4.2	0.9	44.3	33.5	16.8	4.2	1.2	77.8	Strongly agree or agree
	My dog is constantly attentive to me.	4.0	1.0	33.7	39.9	17.8	7.0	1.6	73.6	Strongly agree or agree
	How often do you tell your dog things you don't tell anyone else?	3.7	1.6	50.3	19.3	6.8	1.6	22.0	69.6	Once a day or once a week
	I would like to have my dog near me all the time.	3.9	1.0	35.5	32.3	24.4	6.8	1.0	67.8	Strongly agree or agree
Perceived costs	My dog costs too much money.	2.8	1.0	4.0	21.9	36.7	24.8	12.6	25.9	Strongly agree or agree
	There are major aspects of owning a dog I don't like.	2.4	1.2	4.8	15.1	20.8	34.7	24.6	19.9	Strongly agree or agree
	It is annoying that I sometimes have to change my plans because of my dog.	2.3	1.1	3.6	13.8	24.6	29.7	28.3	17.4	Strongly agree or agree
	How often do you feel that looking after your dog is a chore?	1.8	1.2	4.4	9.8	13.6	9.6	62.6	14.2	Once a day or once a week
	My dog makes too much mess.	2.3	1.1	4.4	9.8	20.8	37.9	27.1	14.2	Strongly agree or agree
	How often does your dog stop you doing things you want to?	2.0	1.2	3.0	9.4	19.5	16.0	52.1	12.4	Once a day or once a week
	It bothers me that my dog stops me doing things I enjoyed doing before I owned it.	2.1	1.1	4.0	8.0	22.2	29.7	36.1	12.0	Strongly agree or agree
	How often do you feel that having a dog is more trouble than it is worth?	1.6	1.1	3.6	5.4	8.8	12.0	70.2	9.0	Once a day or once a week
	How hard is it to look after your dog?	2.2	0.9	1.0	4.8	28.7	40.5	25.0	5.8	Very difficult or difficult

*Within each sub-scale group, items are sorted in descending order of the percentage of respondents scoring 4 or 5*.

Ninety-four point six percent and 88.4% of respondents played games with their dogs or had their dog with them while relaxing at least once a day or once every few days, respectively, and 69.6% of respondents told their dogs things that didn't tell anyone else once a day or at least once a week. Eighty-six-point eight percent of respondents hugged their dogs at least once a day or once every few days, and 80% agreed or strongly agreed with the statement “my dog helps me get through tough times.”

To understand the relationship between perceived costs and the interaction and emotional closeness items, a series of correlations was calculated. An additional measure, “How hard is it to look after your dog?,” was chosen as a factor, because in the convenience sample collected during the COVID lockdown, this item of perceived cost was the only one for which a large percentage of the population reported an increase. The results are summarized in Table 2. In the table, significant correlations are highlighted in bold.

**Table 2:** Correlations between individual MDORS items and the scores for perceived costs and “How hard is it to look after your dog” (representative sample).

				**Perceived costs score**			**“How hard is it to look after your dog?”**		
		**Mean score**	**SD**	**Spearman r**	***P* (two-tailed)**	**95% confidence interval**	**Spearman r**	***P* (two-tailed)**	**95% confidence interval**
Interaction	How often do you play games with your dog?	4.7	0.7	**−0.29**	** <0.0001**	**−0.37 to** **−0.2**	**−0.27**	** <0.0001**	**−0.35 to** **−0.18**
	How often do you have your dog with you while relaxing, ie watching TV?	4.5	1.0	**−0.32**	** <0.0001**	**−0.4 to** **−0.23**	**−0.26**	** <0.0001**	**−0.34 to** **−0.17**
	How often do you hug your dog?	4.5	1.0	**−0.24**	** <0.0001**	**−0.32 to** **−0.15**	**−0.23**	** <0.0001**	**−0.31 to** **−0.14**
	How often do you kiss your dog?	3.7	1.7	**−0.18**	** <0.0001**	**−0.27 to** **−0.1**	**−0.22**	** <0.0001**	**−0.31 to** **−0.13**
	How often do you give your dog food treats?	3.5	1.3	−0.10	0.0201	−0.19 to −0.01	**−0.15**	**0.0005**	**−0.24 to** **−0.06**
	How often do you take your dog to visit people?	3.5	1.5	−0.12	0.0057	−0.21 to −0.03	**−0.16**	**0.0003**	**−0.25 to** **−0.07**
	How often do you groom your dog?	3.4	1.1	−0.06	0.1651	−0.15 to 0.03	**−0.18**	** <0.0001**	**−0.27 to** **−0.09**
	How often do you take your dog in the car?	2.9	1.2	0.01	0.8161	−0.08 to 0.1	−0.12	0.0079	−0.21 to −0.03
	How often do you buy your dog presents?	3.0	1.0	0.00	0.9925	−0.09 to 0.09	−0.06	0.1499	−0.15 to 0.03
Emotional closeness	If everyone else left me my dog would still be there for me.	4.6	0.7	**−0.28**	** <0.0001**	**−0.36 to** **−0.19**	**−0.23**	** <0.0001**	**−0.31 to** **−0.14**
	My dog provides me with constant companionship.	4.6	0.7	**−0.33**	** <0.0001**	**−0.41 to** **−0.25**	**−0.28**	** <0.0001**	**−0.36 to** **−0.19**
	How traumatic do you think it will be for you when your dog dies?	4.4	0.8	**−0.30**	** <0.0001**	**−0.38 to** **−0.22**	**−0.26**	** <0.0001**	**−0.34 to** **−0.17**
	My dog is there whenever I need to be comforted.	4.5	0.8	**−0.28**	** <0.0001**	**−0.37 to** **−0.2**	**−0.29**	** <0.0001**	**−0.37 to** **−0.21**
	I wish my dog and I never had to be apart.	4.4	0.8	**−0.37**	** <0.0001**	**−0.44 to** **−0.28**	**−0.33**	** <0.0001**	**−0.41 to** **−0.25**
	My dog helps me get through tough times.	4.3	0.9	**−0.29**	** <0.0001**	**−0.37 to** **−0.20**	**−0.29**	** <0.0001**	**−0.37 to** **−0.21**
	My dog gives me a reason to get up in the morning.	4.2	0.9	**−0.26**	** <0.0001**	**−0.35 to** **−0.18**	**−0.25**	** <0.0001**	**−0.34 to** **−0.17**
	My dog is constantly attentive to me.	4.0	1.0	−0.11	0.0153	−0.20 to −0.02	**−0.18**	** <0.0001**	**−0.26 to** **−0.09**
	How often do you tell your dog things you don't tell anyone else?	3.7	1.6	−0.08	0.0606	−0.17 to 0.01	**−0.14**	**0.002**	**−0.23 to** **−0.05**
	I would like to have my dog near me all the time.	3.9	1.0	**−0.29**	** <0.0001**	**−0.37 to** **−0.21**	**−0.27**	** <0.0001**	**−0.35 to** **−0.19**

*For convenience of reference, the order of items is as in Table 1. The threshold for significance after Bonferroni correction is p < 0.0026. Significant correlations are highlighted in bold*.

The score for “How hard is it to look after your dog?” was negatively correlated with more of the interaction and emotional closeness items than the overall perceived costs score was. Within the interaction sub-scale, physical contact (hugging and kissing) and shared activities (play and relaxation time together), were both negatively correlated with both perceived costs (Spearman *r* −0.24, −0.18, −0.29, and −0.32, respectively, *p* <0.0001 in all cases) and “how hard is it to look after your dog?” (Spearman *r* −0.23, −0.22, −0.27, and −0.26, respectively, *p* <0.0001 in all cases). Within the emotional closeness sub-scale, only “my dog is constantly attentive to me,” and “how often do you tell your dog things you don't tell anyone else?,” were not correlated with both perceived costs and “how hard is it to look after your dog?.” Although all of these correlations were significant, they were modest in size.

### Spanish COVID Convenience Sample Data

The mean age of the 794 respondents was 40.7 years of age, with 90.2% being female and 9.8% being male. The mean number of dogs per household was 1.6 (SD 1.02). The mean duration of confinement reported by respondents was 3.2 weeks (SD 1.19). Table 3 shows the mean score and standard deviation for each C/DORS item, as well as the results for the single-sample Wilcoxon test. Apart from “How often do you buy your pet presents?,” which was insignificant (*p* = 0.12), all results were significantly different from the mid-point response “The same as before the confinement” at the level of *p* <0.0001. The percentage of respondents giving each response is also presented in Table 3, along with the overall percentages that answered more or much more than before, the same as before, or less or much less than before.

**Table 3:** Individual item scores for C/DORS items and the results of the single-sample Wilcoxon signed rank test (convenience sample, COVID).

				**%**					**% much more or more**	**% same as before**	**% much less or less**	**Wilcoxon signed rank test**	
		**Mean**	**SD**	**2**	**1**	**0**	**−1**	**−2**				**Sum of ranks (W)**	***p***
Interaction	How often do you play games with your dog?	0.66	0.72	8.9	53.0	33.2	4.4	0.5	61.9	33.2	4.9	121,267	<0.0001
	How often do you have your dog with you while relaxing, i.e., watching TV?	0.55	0.73	13.6	28.2	57.8	0.4	0.0	41.8	57.8	0.4	55,596	<0.0001
	How often do you hug your dog?	0.57	0.72	11.1	37.4	49.6	1.5	0.4	48.5	49.6	1.9	74,350	<0.0001
	How often do you kiss your dog?	0.35	0.81	10.3	23.6	59.2	5.0	1.9	33.9	59.2	6.9	35,250	<0.0001
	How often do you give your dog food treats?	0.30	0.64	2.4	31.3	61.0	4.2	1.1	33.7	61.0	5.3	33,529	<0.0001
	How often do you groom your dog?	0.21	0.59	2.4	21.8	70.5	4.5	0.8	24.2	70.5	5.3	17,271	<0.0001
	How often do you buy your dog presents?	0.03	0.58	2.1	10.6	76.7	9.5	1.1	12.7	76.7	10.6	2,100	0.12
Emotional closeness	If everyone else left me, my dog would still be there for me.	0.34	0.66	10.1	14.2	75.1	0.6	0.0	24.3	75.1	0.6	19,106	<0.0001
	My dog provides me with constant companionship.	0.67	0.72	14.9	37.3	47.6	0.2	0.0	52.2	47.6	0.2	86,138	<0.0001
	My dog is there whenever I need to be comforted.	0.31	0.63	8.2	15.5	75.9	0.3	0.1	23.7	75.9	0.4	17,767	<0.0001
	I wish my dog and I never had to be apart.	0.41	0.75	13.4	15.7	69.5	0.9	0.5	29.1	69.5	1.4	26,972	<0.0001
	My dog helps me get through tough times.	0.58	0.73	13.5	31.6	54.3	0.5	0.1	45.1	54.3	0.6	64,423	<0.0001
	My dog gives me a reason to get up in the morning.	0.20	0.56	4.8	12.7	80.4	1.6	0.5	17.5	80.4	2.1	9,667	<0.0001
	My dog is constantly attentive to me.	0.77	0.74	16.5	45.7	36.3	1.4	0.1	62.2	36.3	1.5	123,265	<0.0001
	How often do you tell your dog things you do not tell anyone else?	0.26	0.54	4.6	16.8	78.2	0.3	0.1	21.4	78.2	0.4	14,470	<0.0001
	I would like to have my dog near me all the time.	0.33	0.65	8.0	18.8	71.7	1.4	0.1	26.8	71.7	1.5	23,268	<0.0001
Perceived costs	My dog costs too much money.	−0.05	0.38	0.3	1.5	93.8	1.8	2.6	1.8	93.8	4.4	−749	<0.0001
	There are major aspects of owning a dog I do not like.	−0.27	0.71	0.2	3.1	77.5	7.7	11.5	3.3	77.5	19.2	−13,403	<0.0001
	It is annoying that sometimes I have to change my plans because of my dog.	−0.23	0.63	0.3	1.4	82.7	6.8	8.8	1.7	82.7	15.6	−8,321	<0.0001
	How often do you feel that looking after your dog is a chore?	−0.21	0.62	0.0	2.1	83.8	5.4	8.7	2.1	83.8	14.1	−7,348	<0.0001
	I feel that my dog makes too much mess.	−0.08	0.62	0.9	6.4	82.9	3.5	6.3	7.3	82.9	9.8	−3,724	<0.0001
	How often does your dog stop you doing things you want to?	−0.19	0.59	0.4	2.4	82.0	8.7	6.5	2.8	82.0	15.2	−7,909	<0.0001
	It bothers me that my dog stops me doing things I enjoyed doing before I owned it.	−0.18	0.59	0.5	1.1	86.2	4.8	7.4	1.6	86.2	12.2	−5,041	<0.0001
	How often do you feel that having a dog is more trouble than it is worth?	−0.17	0.64	0.9	3.4	81.6	6.4	7.7	4.3	81.6	14.1	−7,023	<0.0001
	How hard is it to look after your dog	0.25	0.80	5.3	29.0	54.4	8.5	2.8	34.3	54.4	11.3	30,829	<0.0001

*For convenience of reference, the order of items is as in Table 1. The threshold for significance after Bonferroni correction is p < 0.002*.

For the interaction and emotional closeness items, 12.7–62.2% of respondents indicated a change of more or much more during the COVID-19 lockdown, with present-buying for the dog showing the lowest percentage of change and “My dog is constantly attentive to me” the highest. Perceived costs items tended to stay the same or be reduced, with the greatest reported reductions being for “there are major aspects of owning a dog that I don't like,” “It is annoying that sometimes I have to change my plans because of my dog,” and “How often does your dog stop you doing things you want to.” “How hard it is it to look after your dog?” was the only perceived costs item that increased in a large percentage of respondents (34.3%).

Looking at items that are associated with support, 61.9% of respondents played games with their dogs more or much more, 41.8% had their dogs with them while relaxing more or much more, and 48.5% hugged their dog more or much more. Fifty-two point two percent of respondents indicated that their dog provided them with constant companionship more or much more, and 45.1% said that the dog helped them through tough times more or much more.

## Discussion

The context of this paper was the global COVID-19 pandemic and Spanish national confinement lockdown that occurred from 14th March (2020) onward. Many families were isolated at home, people were unable to work, and their households experienced financial, emotional, health and lifestyle impacts. As the pandemic developed, many families also experienced grief.

The Monash Dog Owner Relationship Scale (MDORS) that we used with the representative sample population includes items that measure aspects of social support, as this is one of the benefits proposed as the basis for a functioning relationship in the social-exchange theory (17). In our study we abstracted those social support items to evaluate them outside the subscales that MDORS uses to quantify the human-animal bond. Due to their anonymity, it was not possible for us to re-contact the members of the representative sample panel during the COVID pandemic. Had we collected data from a convenience sample population and compared that with our representative data, differences would have been confounded by demographic biases.

So, when we collected data during the COVID-19 lockdown in Spain we changed the scoring of the scale to a relative measure for each item (a 5-point scale from “much more” to “much less” than before the lockdown). Also, we wanted to look at cat and dog owners, so we used the C/DORS scale that is an adaptation of MDORS for use with both species. C/DORS contains several additonal items on pet-owner interaction that are more specific to cats. However, for the present study the items included are the same for the two populations, aside from those items which were excluded in the Spanish lockdown study because they involved activities that were potentially in breach of the law (such as traveling to other people's homes).

The findings from the lockdown study have already been published (21), but in that paper we gave only a broad overview of the effect of the lockdown on the lifestyle, quality of life and behavior of people and their pets. We found that emotional and lifestyle impacts on the household were those most strongly associated with a perceived negative effect on personal quality of life during the confinement, but we did not look in detail at the data from C/DORS and the support people got from their pets.

The discussion will prioritize the findings from the representative sample population obtained prior to the pandemic in detail and separately from the findings from the COVID-19 lockdown, because it offers a more generalizable insight into the social support people get from their companion dogs. Findings from the sample obtained during the COVID-19 lockdown should be considered only as an illustration of how social support might be realized during a crisis, since, as it is from a convenience sample, the data is exposed to various demographic and recruitment biases.

### Representative Sample Population

Although numerous studies have collected information about the human-animal bond using convenience samples, the results have not been generalizable because of biases in the sex, age and other demographic features of the studied populations (15, 16, 22). To our knowledge, our study is the first to present findings from a representative sample, and as a result we are able to make reasonable generalizations about how valuable dogs are in the social support networks of their owners.

MDORS includes a range of items grouped into sub-scales of owner-dog interaction, emotional closeness and perceived costs. Unlike other measures of the human-animal bond, MDORS has been tested for both reliability and validity (23). It also has widespread use (23), including in studies of dog ownership satisfaction (24) the performance of seizure detection dogs (25), the effects of service dogs on the psychosocial health and well-being of individuals (26), and the effects of dog-owner relationship on perceived stress and happiness (27). Although studies of associations between MDORS and biological measures in dogs and people are limited, there is some evidence of an association between MDORS parameters and plasma oxytocin levels in people and dogs (28).

Some of the MDORS items are more directly relevant to the social support that the owner gets from the dog. These include direct statements about support, such as “My dog helps me get through tough times.” However, MDORS also includes items that do not necessarily relate to support, such as “how often do you take your dog in the car.” For this study we focussed on those items which relate most closely to social support and caregiving.

Table 4 presents those MDORS items which are most relevant to social support, grouped according the previously mentioned characteristics, with a summary of the percentage of respondents who gave the most positive answers.

**Table 4:** Key items from MDORS that relate to social support, with the percentage of respondents who gave the positive responses for each item (representative sample population).

	**% scoring 4 or 5**	**Meaning of score 4 or 5**
**Stated support**		
My dog helps me get through tough times.	80.0	Strongly agree or agree
My dog gives me a reason to get up in the morning.	77.8	Strongly agree or agree
**Availability**		
If everyone else left me my dog would still be there for me.	90.6	Strongly agree or agree
My dog provides me with constant companionship.	90.2	Strongly agree or agree
My dog is there whenever I need to be comforted.	87.0	Strongly agree or agree
My dog is constantly attentive to me.	73.6	Strongly agree or agree
**Shared activity**		
How often do you play games with your dog?	94.6	At least once a day or once every few days
How often do you have your dog with you while relaxing, i.e., watching TV?	88.4	At least once a day or once every few days
**Confidant**		
How often do you tell your dog things you don't tell anyone else?	69.6	Once a day or once a week
**Physical contact**		
How often do you hug your dog?	86.8	At least once a day or once every few days
How often do you kiss your dog?	69.1	At least once a day or once every few days
**Opportunity for care giving**		
How often do you give your dog food treats?	56.8	At least once a day or once every few days
How often do you groom your dog?	54.7	At least once a day or once every few days
How often do you buy your dog presents?	29.2	Once a week or once a fortnight

Two items in MDORS stand out as are direct statements of the degree of support that people get from their dogs; “My dog helps me get through tough times” and “My dog gives me a reason to get up in the morning.” Both of these statements gained a high level of agreement, with 80% of respondents agreeing or strongly agreeing with the former (50.7% strongly agreed with the statement). Lack of motivation is a common characteristic of people who are in an anhedonic or depressive state, so the fact that 77.8% of respondents agreed or strongly agreed with the statement “My dog gives me a reason to get up in the morning” is a powerful indicator of the importance of the human-dog relationship.

However, agreement with direct statements of this kind may not reflect the actual support people get from their dogs. If it is real, the social support people get from their dogs should conform to what we know about the characteristics of social support between people.

### Availability

Availability is one of the main predictors of perceived social support (4). It is worth noting that in Spain, 26% of families live with at least one dog (12), making dogs a readily availability source of social support for many families. Four items from the MDORS refer to the availability of support, and all achieved a high level of agreement from respondents in the representative sample, with 69.8% strongly agreeing with the statement “If everyone else left me, my dog would still be there for me,” 67.2% strongly agreeing with “My dog provides me with constant companionship” and 60.9% strongly agreeing with “My dog is there whenever I need to be comforted.” Of these, the statement about the dog still being there for the person even after everyone else left could be seen as an indication of the emotional support a grieving person might get from their dog. This finding is in agreement with previous research that indicated that living with a dog seems to help people to get through the early stages of bereavement, particularly if the person actively seeks support from the dog (29).

Almost three out of four dog owners in this study declared that their dogs are constantly attentive to them. This proactive attitude perceived by dog owners is consistent with previous research on human-dog interactions, which have found that dogs are able to detect and interpret primary human emotional responses, including expressions of negative affective states (30, 31). For example, dogs proactively approach people more often if they are crying than showing other emotionally neutral vocalizations (32). This ability can be partially explained by selection pressure favoring the survival of dogs that exhibited human-oriented behaviors during the process of domestication (33). Recent evidence suggests that dogs are able not only to express emotional contagion but also to show prosocial helping behaviors, regardless of whether they are truly intentional or only perceived as such by their caretakers (31). In our study, the majority of owners perceived their dogs to be readily available and actively motivated to provide them with emotional support.

### Shared Activities

Playing games with the dog and relaxing with the dog are two important shared activities. More than 90% of participants in the present study said that they play games with their dogs at least once a day or once every few days. Just under 90% of respondents stated that they had their dog with them while they were relaxing at least once a day or once every few days.

Interactive play is considered one of the main benefits of interacting with dogs, together with going on walks (34, 35), and interactive play with dogs seems to be particularly beneficial in promoting prosocial behavior in children (35). The frequency of other shared activities included in the MDORS, such as going with the owner to visit people and on car trips are more influenced by factors outside the relationship, including lifestyle preferences, the dog's dislike of travel or its inability to be left alone at times. In our opinion play and time relaxing are the best overall indicators of the level of shared activities between people and their dogs within MDORS.

### Self-Disclosure and the Confidant

Self-disclosure is the main characteristic of a confidant relationship (9). In this study, the majority of dog owners reported telling their dogs things they didn't tell anyone else at least once a day or once a week (69.6%). This fits with findings from a study comparing self-disclosure to partners and companion dogs, which found that for dog owners the dog played a similar confidant role as the person's partner, with people showing greater willingness to talk to their dog about depression, jealousy, anxiety, calmness, apathy, and fear-related emotions, compared with a human confidant (36).

Although it is easy to be dismissive of this confidant relationship between owner and dog because the dog is incapable of understanding what is being disclosed, evidence from the use of text-based chatbots and artificial intelligence (AI) based counseling systems suggest that people benefit from disclosure even when they know that the counselor is inanimate. The first evidence that computerized chatbots could be convincing conversational partners that were able to elicit disclosure came from the ELIZA studies at MIT (37); one of the response scripts for this system was a convincing simulation of Rogerian psychotherapy (38). More effective chatbots are being developed to use AI to analyze user inputs and patterns of communication in order to respond to human emotions (39), which is something that dogs do naturally. The fact that so many owners regularly engage in personal disclosure to their dogs should be considered an important indicator of the nature of the relationship.

### Physical Contact

Touch and physical contact are important for socio-emotional, physical and psychological well-being (10, 40, 41). In a laboratory study of the effects of physical contact on scores for loneliness, touch contact was found to reduce the perception of loneliness, especially among single people (42). In a study observing the naturally occurring touch contact between married partners whilst they were discussing personal stressors, disclosers who received more touch contact perceived that they were better able to overcome their stressors (43). They also reported greater decreases in self-reported stress, greater increases in self-esteem, and had a more positive view of their partners. Touch may even have effects on immune response that are of relevance to the current pandemic. In a study of the effects of stress-buffering social support and hugging on rate of infection after exposure to the common cold virus, Cohen et al. found that perceived support protected against the rise in infection risk associated with increasing frequency of social conflict, and that 32% of this effect was associated with hugging (44). In a study of psychiatric assistance dogs (PAD) used to help people with mental health disorders, patients were found to make use of tactile and body contact with dogs to help reduce their anxiety (45). A psychiatric assistance dog is a specific type of service dog that has been trained to assist its owner, and in that study PAD owners showed reduce rate of hospitalization and medication use, and an increase in ability to attend appointments.

In the present study, 86.8% of respondents said that they hugged their dog at least once every day or every few days. Likewise, 69.1% of respondents kissed their dog at least once every day or every few days. For people living alone, these may be valuable sources of physical contact. There is also evidence from a study of plasma levels of oxytocin in dogs and their owners during an interaction experiment, that the level of this hormone was associated with increased frequency of kissing the dog (23). During the pandemic, social distancing, restrictions on social interactions between people, and individual concerns about disease transmission have meant that many people have been isolated from sources of physical contact. For many people, this included reducing physical contact with resident friends and family members who worked outside the household in occupations associated with increased disease risk (such as healthcare). However, the social restrictions and isolation that the whole population has experienced during the pandemic is just a taste of what many disadvantaged, elderly people, and people with disabilities or mental illness experience as part of their everyday lives.

### Opportunities for Care Giving

The majority of participants in our study reported that they engaged in activities related to caring for the dog on a daily or nearly daily basis. Helping others, including family members, friends and neighbors, can be as beneficial to health and well-being as receiving support. Caregiving has been associated with psychological benefits for the caregiver, particularly when caring behavior is not perceived as a burden (11).

We also explored the association between perceived costs on aspect of interaction and emotional closeness. Most of the key indicators of social support in MDORS were negatively correlated with perceived costs or difficulty looking after the dog, as shown in Table 5.

**Table 5:** Correlations between key MDORS items associated with social support and perceived costs and stated difficulty with looking after the dog.

	**Correlation (Spearman** ***r*****)**	
	**Perceived costs score**	**“How difficult is it to look after your dog”**
**Stated support**		
My dog helps me get through tough times.	−0.29	−0.29
My dog gives me a reason to get up in the morning.	−0.26	−0.25
**Availability**		
If everyone else left me my dog would still be there for me.	−0.28	−0.23
My dog provides me with constant companionship.	−0.33	−0.28
My dog is there whenever I need to be comforted.	−0.28	−0.29
My dog is constantly attentive to me.		−0.18
**Shared activity**		
How often do you play games with your dog?	−0.29	−0.27
How often do you have your dog with you while relaxing, ie watching TV?	−0.32	−0.26
**Confidant**		
How often do you tell your dog things you don't tell anyone else?		−0.14
**Physical contact**		
How often do you hug your dog?	−0.24	−0.23
How often do you kiss your dog?	−0.18	−0.22
**Opportunity for care giving**		
How often do you give your dog food treats?		−0.15
How often do you groom your dog?		−0.18
How often do you buy your dog presents?		

*Only correlations that were significant after Bonferroni correction are presented (non-significant correlations are left blank)*.

This suggests that increased perceived costs impair the owner's perception of, and ability to use, the dog as a source of support. Interestingly, a time-consuming activity like grooming the dog showed a weak but significant negative correlation with perceived cost, suggesting that it is not generally perceived as a burden by most dog owners who participated in our study.

## Convenience Sample Population (COVID-19 Pandemic)

The COVID-19 pandemic provided an opportunity to gather supporting evidence for the findings from the representative sample population. This was a time of isolation, stress and bereavement that affected entire nations. We would expect people to turn to their dogs for support, and for the relevant key measures of MDORS to be increased. This is what we found (see Table 6). The average time of confinement was 3.2 weeks, which may be regarded as quite short. However, previous studies indicate that periods of quarantine and home confinement as short as 10 days have been associated with negative psychological consequences (46).

**Table 6:** Percentage of respondents in the COVID convenience sample who rated key MDORS items as much more, more or the same as before the lockdown.

	**% Much more or more**	**% Same as before**	**% More or same as before**	**Mean % reporting an increase**
**Stated support**				
My dog helps me get through tough times.	45.1	54.3	99.4	31.3
My dog gives me a reason to get up in the morning.	17.5	80.4	97.9	
**Availability**				
If everyone else left me my dog would still be there for me.	24.3	75.1	99.4	40.6
My dog provides me with constant companionship.	52.2	47.6	99.8	
My dog is there whenever I need to be comforted.	23.7	75.9	99.6	
My dog is constantly attentive to me.	62.2	36.3	98.5	
**Shared activity**				
How often do you play games with your dog?	61.9	33.2	95.1	51.9
How often do you have your dog with you while relaxing, i.e., watching TV?	41.8	57.8	99.6	
**Confidant**				
How often do you tell your dog things you don't tell anyone else?	21.4	78.2	99.6	21.4
**Physical contact**				
How often do you hug your dog?	48.5	49.6	98.1	41.2
How often do you kiss your dog?	33.9	59.2	93.1	
**Opportunity for care giving**				
How often do you give your dog food treats?	33.7	61.0	94.7	23.5
How often do you groom your dog?	24.2	70.5	94.7	
How often do you buy your dog presents?	12.7	76.7	89.4	

*Mean percentage reporting an increase for each groups of items*.

Increases were seen in each of the key areas of social support, but were greatest for shared activity, physical contact and availability. Interestingly, these would be the areas of human social interaction that would seem to be the most affected by the pandemic confinement, which implies that the social support provided by the dog dynamically adapted to fill gaps in social interaction and support created by the lockdown.

Forty-five-point one percent of respondents said that their dog helped them through tough times more or much more than before the confinement. There were increased ratings for every one of the key indicators, with “my dog is constantly attentive to me,” “How often do you play games with your dog,” “How often do you hug your dog,” and “My dog provides me with constant companionship” being increased the most. However, “How often do you tell your dog things that you don't tell anyone else” was increased for only 21.4% of respondents, and this may reflect the fact that the use of video-conferencing technology has enabled many people to maintain a degree of communication with their human confidants.

Perceived costs items tended to stay the same or be reduced, which is unsurprising given that the data was collected during the lockdown; a time when having a dog would be expected to have less of an impact on owners' plans and activities. Although we would expect that the lockdown would have imposed a significant financial burden on households, the level of agreement with the statement “My dog costs too much money” hardly changed.

## Limitations of this Study and Future Work

Using a representative sample population we were able to make a general characterization of the social support people get from dogs, and find out how widespread it is. However, we were unable to follow-up the same population during the pandemic, so we had to adapt a version of the same scale to provide a relative measure for each item, and we collected data from a convenience sample. These were compromises that we felt were worthwhile, given the opportunity to collect data during such an unusual event, but they limit the value of the lockdown data. Future studies should address these limitations. During the lockdown we also did not explore the relationship between individual circumstances and the social support obtained. For example, how the type and level of social support from the dog related to the quality of a person's wider social support network, the stresses the person experienced, their physical and mental health, and how technological solutions (such as video calling and social media) had mitigated the non-physical aspects of social isolation. These are areas that require further study.

## Practical Implications

Our findings indicate that dog owners treat their dogs as a source of social support that offers a unique combination of characteristics; emotional responsiveness, physical contact, being non-judgmental and unquestioning in the face of personal disclosure, and apparently unaffected by the underlying causes of the person's distress. In addition, what is qualitatively lacking in each of these characteristics, when compared with the support that might be provided by a person, may be made up for by the immediacy of availability.

Our data from the COVID-19 pandemic show that dogs can be a valuable source of social support during a time of crisis, and in particular a source of physical contact to people during a time of social isolation.

This suggests that the social support dogs provide should be factored into public health decisions about how to manage crises, such as a pandemic, and interventions to help people who are in distress. This could include interventions to take advantage of the presence of a dog in the household as part of psychological or counseling interventions or finding ways to alleviate perceived costs and difficulties of ownership that we have shown can impair perceived support from the dog in a number of important areas.

For example, if we want dog owners to gain the maximum benefit from having a dog during a period of personal crisis, interventions could be targeted to make owning the dog easier. On an individual basis, this could include helping with the costs of food and veterinary care or helping a person who is elderly or has a disability to exercise their dog. During a national emergency, such as the COVID-19 lockdown, this could include making public announcements that the availability of dog food and healthcare will not be affected, and that dogs will continue to be able to go outside (as we found in our previous study that these were specific concerns dog owners had).

Hodgson et al. identified 4 ways in which pets could benefit human health; as builders of social capital, as agents of harm reduction, as motivators for healthy behavior change, and as potential participants in treatment plans (47). They suggested that pets could motivate regular exercise, encourage activities of daily living, catalyze social interactions and a sense of community, encourage harm reduction (e.g., quitting smoking), and augmenting plans for the treatment of chronic disease. The simplest way to activate these benefits during healthcare interventions would be to ask people about their pets, as this is a non-challenging way to open a dialogue and discover details about a person's social support network, and lifestyle.

The authors of that paper went on to test this hypothesis using primary healthcare providers (PHPs; family physicians, nurses, and social workers) who were trained to include communication with patients about their pets as part of their service. As hypothesized, this was found to be a good way to open up communication that had positive effects on practice and relationships with patients (48). Patients responded quickly and openly to questions about their pets, and this enabled PHPs to learn more about aspects of lifestyle such as physical activity, as well as about patients' family members, social capital and housing. PHPs were also able to use discussion about pets to leverage improvements in social capital, physical exercise, controlling unhealthy behaviors and the therapeutic benefits of pets.

Talking with patients about their pets as a way to improve communication, establish rapport, gather information and leverage support and behavioral change could be of increased importance in situations in which the patient has limited mobility or access to social capital, as we have seen during the pandemic confinement.

## Conclusion

The findings from the representative sample population indicate that the majority of dog owners feel that their dogs help them through tough times, and that the support they get conforms to known characteristics of social support such as availability, shared activity, physical contact and acting as a confidant. In the convenience sample pandemic population, shared activity, physical contact and availability items were, in decreasing order, those which were most increased. The owner-dog relationship therefore seems to be highly adaptable to compensate for changes in other sources of social support. If we want to take make the most of these benefits, we have two options; firstly to create interventions that take advantage of the presence of a dog in the household, and secondly to identify and minimize perceived costs for the owner so that the dog can be most effective as a social support.

## Data Availability Statement

The raw data supporting the conclusions of this article will be made available by the authors, without undue reservation.

## Ethics Statement

The studies involving human participants were reviewed and approved by Social Sciences Research Ethical Review Board (SSRERB) at the Royal Veterinary College. The patients/participants provided their written informed consent to participate in this study.

## Author Contributions

JB and JF designed the study, performed the analysis and drafted the paper. AB assisted with the design of the study, interpretation of the results and final drafting of the paper. All authors contributed to the article and approved the submitted version.

## Conflict of Interest

The authors declare that the research was conducted in the absence of any commercial or financial relationships that could be construed as a potential conflict of interest.
